# On the Curative Treatment of Aneurism of the Aorta

**Published:** 1852-11

**Authors:** O. B. Bellingham


From the Dublin Medical Press.

*On the Curative Treatment of Aneurism of the Aorta, by* O. B. Bellingham, M. D.

At the meeting of the Surgical Society of Ireland, held on the 24th of January, Dr. Bellingham read a most important paper on this subject, and illustrated his observations by cases. The following are the conclusions which the author deduced:

1st. That aneurism of the aorta is not necessarily an incurable disease.

2d. That it appears to be more amenable to curative treatment than is ordinarily supposed.

3d. That treatment ought always to be specially directed to this object.

4th. That when a spontaneous cure occurs, it is always by the gradual deposition of the fibrine of the blood in layers within the aneurismal sac, until it is filled up.

5th. That if we hope to succeed in effecting a cure, it must be by imitating the mode by which nature brings this about.

6th. That in order to favor the gradual deposition of fibrine, we should aim at diminishing the mass of blood, and lessening the strength and rapidity of the current through the aneurismal sac.

7th. That this can only be indirectly accomplished by acting on the general circulation.

8th. That neither bleeding, purgatives, diuretics, digitalis, nor the various other remedies which have been employed in this disease, can be depended upon for producing these effects.

9th. That an extremely restricted diet, particularly in fluids, continued for a certain time, appears to have the effect of rendering the pulse small, compressible and slow, and at the same time, of diminishing the mass of blood.

10th. That the cases related afford evidence that these results may be brought about by treatment conducted on the foregoing plan.

11th. That this method of treatment, to prove effectual, must be steadily and perseveringly carried out, and must be continued until a decided impression is made upon the disease.

12th. That it is adapted, not only to aneurism of the thoracic and abdominal aorta, but to aneurism in any of the immediate branches of these vessels; and that, if employed as a preliminary to compression, pain will be diminished, and the duration of the treatment considerably abridged.

